# Dimeric Structure of the Pseudokinase IRAK3 Suggests an Allosteric Mechanism for Negative Regulation

**DOI:** 10.1016/j.str.2020.11.004

**Published:** 2021-03-04

**Authors:** Sven M. Lange, Marina I. Nelen, Philip Cohen, Yogesh Kulathu

**Affiliations:** 1 MRC Protein Phosphorylation and Ubiquitylation Unit, Sir James Black Centre, Dow Street, Dundee, Scotland DD1 5EH, UK; 2 Discovery, Janssen Research and Development, Welsh and McKean Roads, Spring House, PA 19477, USA

**Keywords:** innate immunity, pseudoenzymes, kinase, signal transduction, protein phosphorylation, IRAK kinases, Myddosome, Toll-like/Interleukin-1 receptor signalling, kinase inhibitors, asthma

## Abstract

Interleukin-1 receptor associated kinases (IRAKs) are key players in innate immune signaling that mediate the host response to pathogens. In contrast to the active kinases IRAK1 and IRAK4, IRAK2 and IRAK3 are pseudokinases lacking catalytic activity and their functions are poorly understood. IRAK3 is thought to be a negative regulator of innate immune signaling and mutations in IRAK3 are associated with asthma and cancer. Here, we report the crystal structure of the human IRAK3 pseudokinase domain in a closed, pseudoactive conformation. IRAK3 dimerizes in a unique way through a head-to-head arrangement not observed in any other kinases. Multiple conserved cysteine residues imply a potential redox control of IRAK3 conformation and dimerization. By analyzing asthma-associated mutations, we identify an evolutionarily conserved surface on IRAK3 that could form an interaction interface with IRAK4, suggesting a model for the negative regulation of IRAK4 by IRAK3.

## Highlights

• The crystal structure of IRAK3 reveals a closed “active-state” conformation
• The pseudokinase domain of IRAK3 dimerizes via a unique head-to-head interface
• Conserved cysteines suggest redox-regulation of IRAK3 conformation and dimerization
• Asthma-associated mutations in IRAK3 locate to putative IRAK4 interaction interface

## Graphical Abstract

**Figure:**
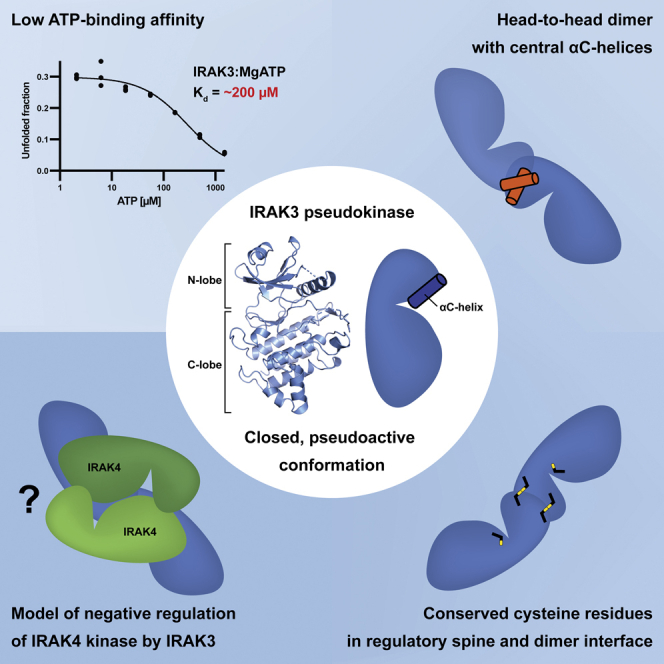

## Teaser

Lange et al. report the first structure of the IRAK3 pseudokinase domain, which reveals IRAK3 to be in a pseudoactive conformation and to form a novel head-to-head dimer. Asthma-associated mutations and structural analyses suggest new mechanistic models on how IRAK3 might function as a negative regulator of innate immunity.

## Introduction

The innate immune system is the first line of defense against bacteria, viruses and parasites. Molecules associated with invading pathogens are recognized by pattern recognition receptors, such as Toll-like receptors (TLRs), initiating a rapid response (Kimbrell and Beutler, 2001). Engaged TLRs recruit the adaptor protein MyD88 (myeloid differentiation primary response protein 88) that serves as an assembly platform for members of the interleukin-1 receptor associated kinase (IRAK) family to form an oligomeric signaling complex called the Myddosome (Motshwene et al., 2009; Lin et al., 2010). Myddosome assembly initiates downstream signaling cascades that trigger the expression and secretion of pro-inflammatory and anti-inflammatory mediators to shape the immune response to pathogens (Winston et al., 1999; Dumitru et al., 2000; Kanayama et al., 2004; Xia et al., 2009; Pauls et al., 2012; Lopez-Pelaez et al., 2014; Cohen and Strickson, 2017).

**Figure 1:**
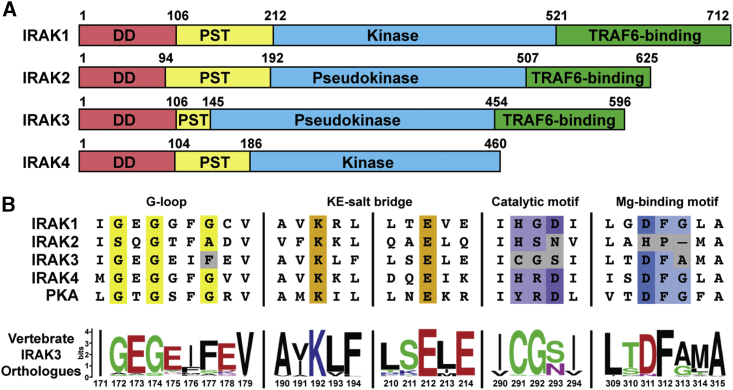
Domain Architecture and Active Site Motifs of the IRAK Family (A) Domain overview of human IRAK family members with indicated domain boundaries. Death domains (DD) in red, Pro-Ser-Thr-rich regions (PST) in yellow, kinase domains and pseudokinase domains in blue, and C-terminal TRAF6-binding regions in green. (B) Sequence alignment of kinase active site motifs in human IRAK family members and PKA with critical residues highlighted in color and deviations from canonical motifs in gray. Below, the consensus sequence of 134 IRAK3 orthologues in vertebrates with polar residues in green, non-polar residues in black, basic residues in blue and acidic residues in red.

In humans, the IRAK family of kinases consists of four members that share a similar domain architecture (Figure 1A). All IRAKs have N-terminal death domains (DDs) of ∼100 amino acids that facilitate oligomerization with the DDs of MyD88 molecules to form the Myddosome (Feinstein et al., 1995; Motshwene et al., 2009). The DDs are followed by stretches of 40 to 100 amino acids rich in proline, serine and threonine residues (PST regions) that undergo hyperphosphorylation during innate immune signaling (Kollewe et al., 2004). The PST region is followed by the respective kinase or pseudokinase domains, and three of the four IRAK family members, IRAKs-1, -2 and -3, have an additional C-terminal domain that contains conserved TRAF6-binding motifs (Ye et al., 2002).

Eukaryotic protein kinases have a common structural fold, consisting of a β-strand-rich N-lobe and a mostly α-helical C-lobe (Taylor and Radzio-Andzelm, 1994) with the ATP-binding pocket and key catalytic residues located between the two lobes. Most canonical protein kinases can switch between a catalytically productive, closed conformation and an unproductive, open conformation. Hallmarks of an active kinase conformation include an inward-oriented αC-helix, the assembly of a hydrophobic regulatory spine (R-spine) and an inward-position of the DFG-motif residues (Kornev et al., 2006; Taylor and Kornev, 2011; Modi and Dunbrack, 2019).

Eukaryotic protein kinases share canonical active site motifs that fulfill critical roles in ATP-binding, Mg^2+^-binding and catalysis of the phospho-transfer reaction (Hanks et al., 1988; Zheng et al., 1993; Taylor and Radzio-Andzelm, 1994; Johnson et al., 1996; Scheeff et al., 2009). The glycine-rich G-loop with the conserved “[G/A/S]xGxx[G/A/S]” sequence motif assists in ATP-binding, while the KE-salt bridge, formed by K72 and E91 in the canonical cAMP-dependent protein kinase catalytic subunit α (PKA), stabilizes the closed, active kinase conformation and positions the lysine for binding of the α-phosphate of ATP. The catalytic “[H/Y]RD”-motif harbors a critical aspartate residue that primes the hydroxyl-group of the substrate for nucleophilic attack on the γ-phosphate of ATP. A second conserved aspartate residue in the “DFG”-motif binds to Mg^2+^-ions of Mg-ATP, positioning and polarizing the γ-phosphate of ATP for efficient transfer onto substrates.

Interestingly, about 10% of the proteins in the human kinome lack one or more critical residues that are required for ATP-binding or for catalysis of the phosphoryl-transfer reaction and are therefore referred to as pseudokinases (Manning et al., 2002; Boudeau et al., 2006; Ribeiro et al., 2019). Detailed examination of individual pseudokinases revealed that they often function as crucial components of cell signaling pathways through unique and novel mechanisms. Pseudokinases can be broadly categorized into three types that (1) retain their catalytic activity by compensatory mechanisms (e.g., other residues substitute for the lack of a canonical active site motif) (Xu et al., 2000; Min et al., 2004; Villa et al., 2009), (2) use alternative catalytic mechanisms to achieve phosphoryl-transfer or even developed entirely different enzymatic activities (Mukherjee et al., 2008; Shi et al., 2010; Sreelatha et al., 2018; Black et al., 2019), and (3) fulfill important non-enzymatic functions including the allosteric regulation of active kinases or as scaffolds that tether interacting partners to larger complexes (Scheeff et al., 2009; Zeqiraj et al., 2009a; Bailey et al., 2015; Lavoie et al., 2018).

IRAK1 and IRAK4 are catalytically active members of the IRAK family (Cao et al., 1996; Li et al., 2002; Motshwene et al., 2009; Flannery and Bowie, 2010). DD-mediated oligomerization of multiple IRAK4 molecules induces *trans*-autophosphorylation of IRAK4 at T345 and S346, which leads to an increase in IRAK1 kinase activity, possibly due to allosteric activation of IRAK1 following binding to phosphorylated IRAK4 (Ferrao et al., 2014; Vollmer et al., 2017). In contrast, IRAK2 and IRAK3 are thought to be catalytically inactive pseudokinases as they both lack the catalytic aspartate and other essential active site residues (Figure 1B) (Muzio et al., 1997; Wesche et al., 1999). Despite being discovered more than two decades ago, the detailed roles of the IRAK2 and IRAK3 pseudokinases are still largely unknown.

IRAK3 is expressed in several tissues and immune cells with highest expression in myeloid cells (Wesche et al., 1999; Lizio et al., 2015). Several lines of evidence suggest that IRAK3 is a negative regulator of signaling, since there is an enhanced inflammatory response to bacterial infection and increased susceptibility to LPS-induced septic shock in *IRAK3*^*−/−*^ mice (Kobayashi et al., 2002). However, many findings about the role of IRAK3 need to be critically re-evaluated following the recent discovery that a widely used *IRAK3*^*−/−*^ mice line may still express a splice variant of IRAK3 (Rothschild et al., 2017) that lacks the C-lobe of the pseudokinase domain and the TRAF6-binding region, yet appears to strongly activate NF-κB-dependent gene transcription.

Nonetheless, clinical data support a role for IRAK3 as a negative regulator of innate immunity, as multiple mutations in IRAK3 have been linked to the pathogenesis of early-onset persistent asthma (Balaci et al., 2007) and high expression levels of IRAK3 and mutations in the pseudokinase domain correlate with an increased risk of cancer (Saenger et al., 2014; Kesselring et al., 2016). While it is unclear how IRAK3 exerts its negative regulatory effects, these findings highlight the potential of IRAK3 as a drug target in immunodeficiency diseases and in cancer immunotherapies.

The crystal structures of the kinase domains of IRAK1 and IRAK4 in complex with various ligands and inhibitors have provided valuable insights into their mechanisms of action (Wang et al., 2006, 2017, 2019; Kuglstatter et al., 2007; Ferrao et al., 2014). In contrast, no structures of the pseudokinase domains of IRAK2 or IRAK3 have been reported to date, and hence their functions and mechanistic details remain elusive.

Here we report the crystal structure of the pseudokinase domain of human IRAK3 at 2.9 Å resolution, which reveals a closed, pseudoactive conformation with an evolutionarily conserved cysteine residue located in the regulatory spine (R-spine). IRAK3 dimerizes in a pseudosymmetric “head-to-head” assembly with central αC-helices, stabilized by two disulfide bridges. We further identify a conserved C-lobe surface on IRAK3 that harbors asthma-associated mutations and closely resembles the previously reported IRAK4 homodimer interface. We propose a model for hetero-oligomerization between IRAK3 and IRAK4 via this C-lobal interface.

## Results

### Sequence Analysis of IRAK3

Sequence alignment of the four human IRAK family members with the canonical kinase PKA, revealed that the canonical kinase active site motifs are conserved in the catalytically active family members, IRAK1 and IRAK4, but not in the pseudokinases IRAK2 and IRAK3 (Figure 1B). While the residues of the G-loop and KE-salt bridge are conserved in IRAK2, the catalytic aspartate is replaced by an asparagine. Interestingly, the Mg^2+^-coordinating “DFG”-motif is changed to “HP,” which suggests an unusual nucleotide-binding mechanism, as IRAK2 has been previously shown to bind ATP in a Mg^2+^-dependent manner (Murphy et al., 2014). In the absence of an IRAK2 structure, we speculate that the basic histidine residue may replace one of the two Mg^2+^-ions in the ATP-binding pocket, reminiscent of the ATP-binding mechanism of the Ca^2+^/calmodulin-activated serine/threonine kinase (CASK) where an analogous histidine residue directly binds ATP (Mukherjee et al., 2008).

IRAK3 is found throughout the *vertebrata* subphylum and a conservation analysis of the primary sequences of 134 vertebrate IRAK3 orthologues shows that several alterations to canonical kinase active site motifs are preserved throughout evolution (Figure 1B). The large aromatic phenylalanine (F177) residue disrupts the G-loop motif at the position of the third glycine and shows strong conservation. The canonical catalytic “HRD”-motif is missing entirely in IRAK3 and replaced with “CGS,” whereas the Mg^2+^-coordinating “DFG”-motif is slightly altered to “DFA.” Interestingly, the “CGS” sequence that replaces the catalytic motif in IRAK3 is also well conserved at C291 and G292, while the position of S293 is frequently occupied by S or N residues in other vertebrates. The Mg-binding loop motif also displays a strong conservation of D311 and F312, while position 313 is primarily occupied by either A or G. However, the residues of the KE-salt bridge are conserved, as they are for all human IRAK family members. These analyses indicate an evolutionarily conserved function for the altered kinase active site residues of IRAK3.

### Crystal Structure of IRAK3 Reveals Closed Conformation

**Figure 2:**
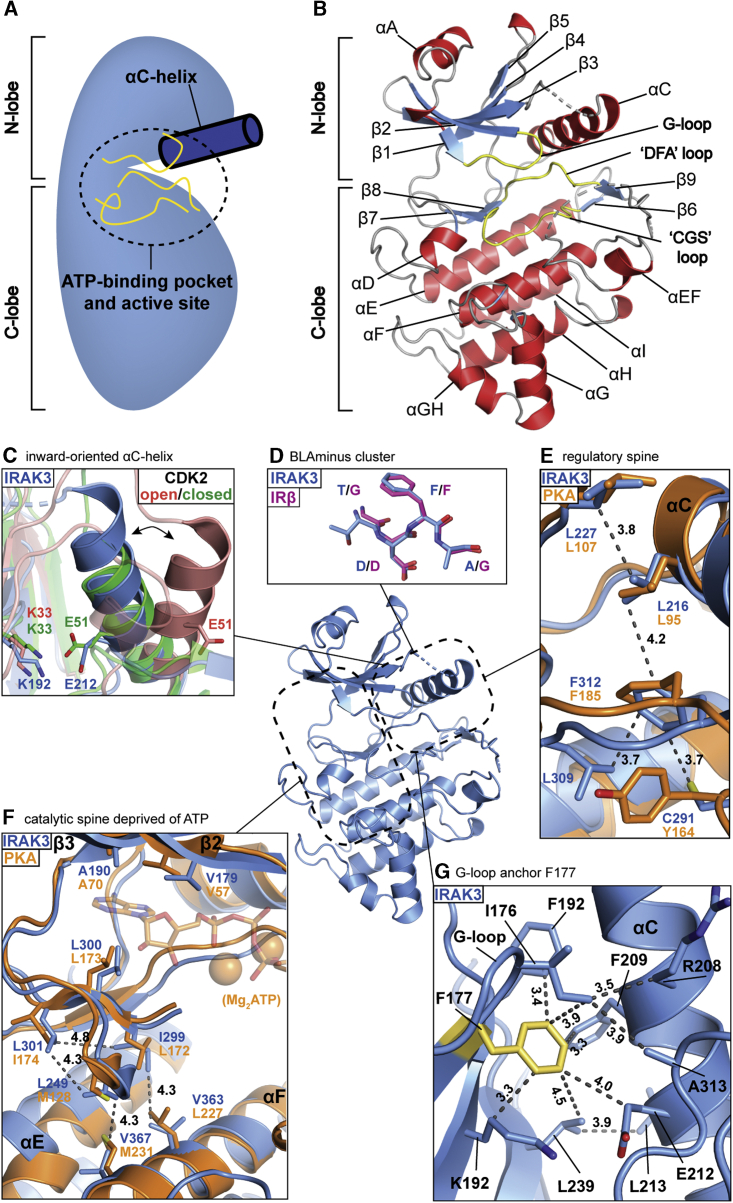
Structure of the IRAK3 Pseudokinase Domain (A) Schematic representation of the eukaryotic protein kinase architecture. (B) Crystal structure of the pseudokinase domain of IRAK3 colored by secondary structure with α-helices in red, β-strands in blue, loops in gray and active site loops in yellow. Dotted lines indicate flexible loops without structural information. (C) IRAK3 (blue) superposed with inactive (red, PDB 3PXF; Betzi et al., 2011) and active (green, PDB 3QHW; Bao et al., 2011) conformations of CDK2. KE-salt bridge residues are shown as stick models. (D) DFA motif of IRAK3 (blue) aligned with DFG motif of IRβ of BLAminus cluster (pink, PDB 3BU5; Wu et al., 2008). (E) Superposition of IRAK3 in blue and PKA in orange (PDB 2CPK) with regulatory spine residues shown as stick models. (F) Superposition of IRAK3 in blue and PKA in orange (PDB 2CPK) with catalytic spine residues shown as stick models. ATP and Mg-ions are shown as semi-transparent stick and sphere models, respectively. Parts of the Mg-binding loops and αD-helices where clipped for visibility. (G) Interaction network formed by hydrophobic anchor residue F177 (yellow) with surrounding residues in IRAK3 (blue) shown as stick models. Gray-dotted lines indicate distance measurements in Å.

To gain more insight into the functions of IRAK3, we crystallized the pseudokinase domain (residues 145–454). The structure was determined at 2.9 Å resolution by molecular replacement and refined to the statistics shown in Table S1. The asymmetric unit (ASU) contains 3 molecules of IRAK3. The structure reveals that the eukaryotic kinase fold is conserved in the pseudokinase domain of human IRAK3 (Figures 2A and 2B). The N-lobe consists of an anti-parallel β-sheet formed by β-strands β1-β5, which are flanked by the αA- and αC-helices. The atypical G-loop of IRAK3 is located between β-strands β1 and β2. The two lobes are bridged by a hinge-region formed by β7, β8 and αD. The “catalytic”-loop with the degenerate “CGS”-motif of IRAK3 connects β6 and β7, while the Mg-binding loop with the “DFA”-sequence resides between β8 and β9. The remaining C-lobe is made up of five tightly packed α-helices (αE, αF, αG, αH and αI) and two short helical extensions (αEF and αGH).

In the crystal structure, IRAK3 adopts a pseudoactive conformation that mimics the active state of canonical protein kinases. The αC-helix of IRAK3 is in an inward-oriented position with an intact KE-salt bridge formed by K192 of the β3-strand and E212 of the αC-helix. Superposition of IRAK3 with structures of active and inactive forms of the archetypal kinase Cyclin-Dependent Kinase 2 (CDK2) illustrates that IRAK3 closely resembles the active conformation (Figure 2C). A recently introduced, improved nomenclature to typify protein kinase conformations is based on the conformation of the DFG motif, the Ramachandran regions they fall in, the backbone dihedral angles of the residue preceding the DFG motif and the side chain rotamer of the Phe residue (Modi and Dunbrack, 2019). Analyzing the equivalent “DFA” motif of IRAK3 reveals that IRAK3 can be assigned to the BLAminus cluster as residue T310, preceding the “DFA”-sequence of IRAK3, falls into the β-sheet region (B), D311 into the left-handed helical region (L) and F312 into the α-helical region (A) of the Ramachandran plot, while the side chain of F312 is close to a −60° rotamer (“minus”). Interestingly, the DFG motif of most active protein kinase conformations fall into the same “BLAminus” cluster. Structural superposition of the DFA motif of IRAK3 with the Mg-binding motif of another BLAminus cluster member, the β-subunit of the insulin receptor kinase (IRβ), presents a well-aligned peptide backbone and phenylalanine side chain reflected by an all-atom RMSD of 0.38 Å^2^ (Figure 2D).

Another hallmark of an active kinase conformation in IRAK3 is the intact regulatory spine (R-spine) formed by L227, L216, F312, C291, and L309 (Figure 2E). The first three R-spine residues of IRAK3 (L227, L216, and F312) match the respective amino acids of the canonical protein kinase PKA (L106, L95 and F185) with an all-atom RMSD of 0.70 Å^2^ (Knighton et al., 1991). The position of Y164 of PKA is occupied by two residues in IRAK3, C291 of the conserved “CGS” sequence and L309, located in the adjacent β8-strand. The presence of this evolutionarily conserved cysteine residue in the R-spine of IRAK3 suggests the possibility of a redox-based mechanism that controls R-spine assembly. In this scenario, the modification or oxidation of C291 would prevent R-spine assembly and thereby stabilize the open pseudokinase conformation of IRAK3. Interestingly, a similar redox-based mechanism has been recently shown to regulate the kinase activity of Aurora A through reversible oxidation of a cysteine in the activation segment (Byrne et al., 2020). In a sequence analysis of the human kinome we identified only one other protein with a cysteine at this position, namely the poorly characterized pseudokinase RPS6KC1 (Ribosomal protein S6 kinase delta-1).

Intriguingly, the structure of IRAK3 also revealed a pre-arranged catalytic spine (C-spine) deprived of ATP. The C-spine is a hydrophobic network formed in an active conformation in canonical kinases, which spans from the central C-lobal αF-helix to the β2-and β3-strands of the N-lobe and is completed by the adenine ring of ATP (Kornev et al., 2008). Superposition of IRAK3 and ATP-bound PKA in an active conformation shows that IRAK3 has equivalent hydrophobic residues (V179, A190, L249, I299, L300, L301, V363, and V367) positioned close to the C-spine residues of PKA (V57, A70, M128, L172, L173, I174, L227, and M231), although IRAK3 is not bound to ATP (Figure 2F). Since the C-spine is already pre-formed in the absence of ATP, we predict that the ATP-bound form of IRAK3 would likely adopt a similar conformation to the nucleotide-free IRAK3 structure as no major structural rearrangements would be necessary to complete the C-spine.

### A Hydrophobic G-loop Anchor Stabilizes the Closed Conformation in IRAK3

**Figure 3:**
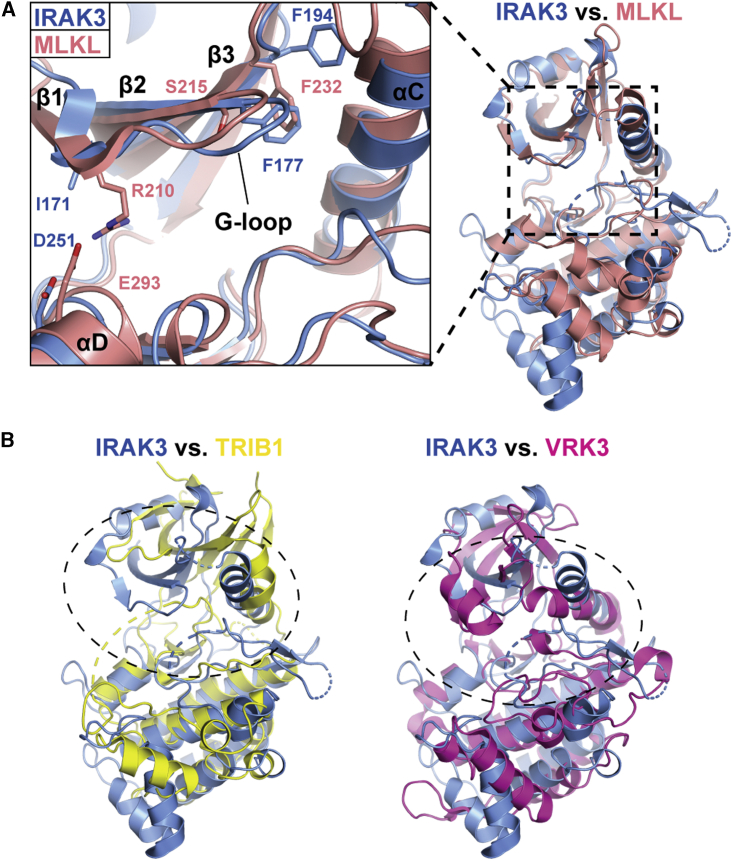
Active Site Comparison between IRAK3 and Other Pseudokinases (A) Superposition of IRAK3 (blue) with the pseudokinase MLKL (salmon, PDB 4WMI; Murphy et al., 2014) and close-up view of the G-loop stabilizing residues shown as stick models. (B) Superposition of IRAK3 (blue) with the pseudokinases TRIB1 (yellow, PDB 5CEM; Murphy et al., 2015) and VRK3 (magenta, PDB 2JII; Scheeff et al., 2009). Dotted circles indicate the severely altered ATP-binding pockets of TRIB1 and VRK3.

The G-loop of IRAK3 contains a degenerate motif of “GEGEIF”, while the active family members, IRAK1 and IRAK4, both have “GEGGFG” at this position. Closer inspection of the G-loop interactions in the IRAK3 structure revealed that F177 anchors the G-loop to residues of the αC-helix, β3-and β4-strands and to the Mg-binding loop (Figure 2G). F177 forms a hydrophobic network with the neighboring I176, with K192 and F194 of the β3-strand, R208, F209, E212, and L213 of the αC-helix, L239 of the β4-strand and A313 of the Mg-binding loop, to stabilize an ordered G-loop. The hydrophobic anchor F177 thereby leads to a rigidification of the G-loop and keeps the loop ordered in the apo-form of IRAK3. This is in contrast to the apo-structures of other protein kinases, where the G-loop is typically disordered and a ligand in the ATP-binding pocket is required to stabilize the loop. The phenylalanine residue in this position of the G-loop is evolutionarily conserved in vertebrate IRAK3 orthologues and appears to be unique within the human kinome (Figure 1B). Interestingly, analysis of the G-loop of the pseudokinase MLKL reveals it to be similarly stabilized in the absence of nucleotides or inhibitors. While MLKL does not possess an equivalent residue to the hydrophobic anchor F177 of IRAK3, the G-loop of MLKL is instead stabilized by a salt bridge formed between R210 of the N-lobal β1-strand and E293 of the C-lobal αD-helix (Figure 3A). In contrast, other pseudokinases harbor more drastic alterations that stabilize a specific conformation. The pseudokinase TRIB1, for example, has a severely truncated G-loop and αC-helix while another pseudokinase, VRK3, has multiple amino acid substitutions in the nucleotide-binding pocket that not only block ATP-binding but also lock the pseudokinase in a closed, pseudoactive conformation (Scheeff et al., 2009) (Figure 3B). In summary, IRAK3 adopts a closed pseudoactive conformation that is stabilized by a network of hydrophobic interactions through the G-loop anchor F177.

### IRAK3 Has Low Affinity for ATP but High Affinity for ATP-Competitive Inhibitors

**Figure 4:**
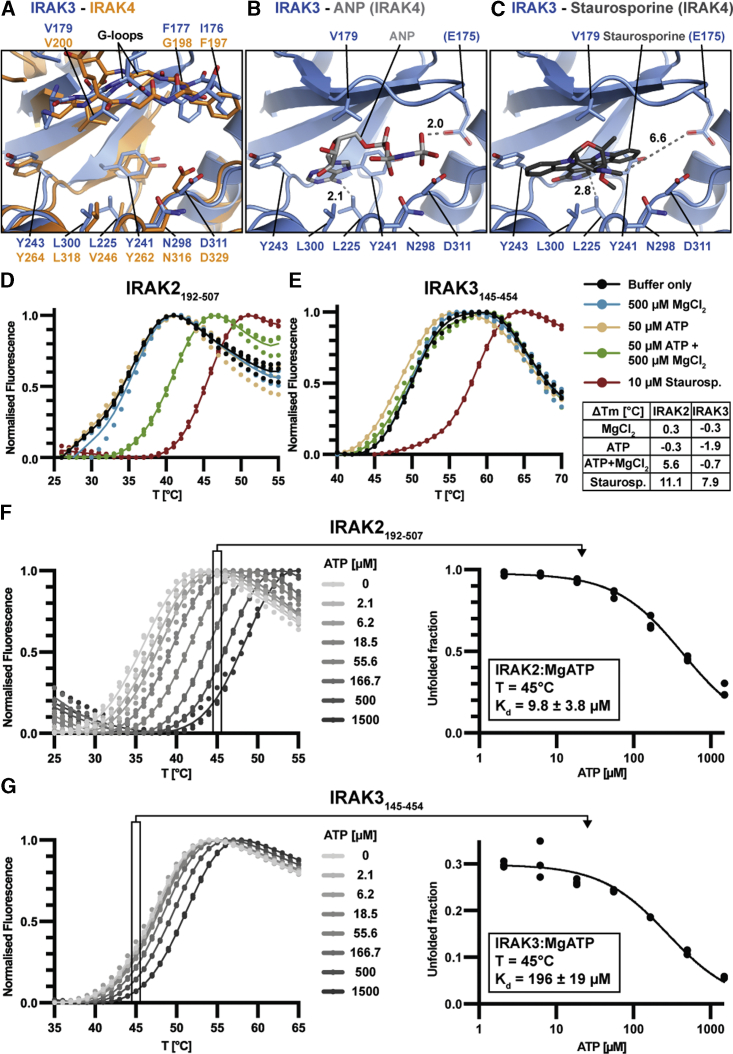
Nucleotide-Binding Analysis of IRAK2 and IRAK3 (A–C) Superposition of ATP-binding pockets of IRAK3 and (A) IRAK4 (PDB 2OID). (B) IRAK4-bound ANP (PDB 2OID) and (C) IRAK4-bound Staurosporine (PDB 2OIC). (D and E) Thermal shift assays of IRAK2_192-507_ (D) and IRAK3_145-454_ (E) with buffer control (black), 500 μM MgCl_2_ (blue), 50 μM ATP (yellow), 50 μM ATP +500 μM MgCl_2_ (green) or 10 μM Staurosporine (red). Table lists induced thermal shifts. (F and G) Thermal shift analaysis of IRAK2_192-507_ (F) and IRAK3_145-454_ (G) at indicated ATP concentrations and 5 mM MgCl_2_ and isothermal analysis of ATP-dose response thermal shift assays at 45°C. Triplicate measurements, dissociation constants (K_d_) are shown with standard error of the mean.

The ATP-binding pocket of IRAK3 is unobstructed in the ligand-free structure and superposition with IRAK4 revealed that most residues involved in ATP-binding in IRAK4 are identical in IRAK3, apart from L225 and E175 (Figure 4A). L225, which is neighboring the tyrosine gatekeeper residue in IRAK3, protrudes further into the ATP-binding pocket compared to V246, the equivalent residue in IRAK4. Superposition of IRAK3 with IRAK4 bound to the ATP-analogue Adenosine-5ʹ-[(β,γ)-imido]-triphosphate (AppNHp) indicates a steric clash of L225 of IRAK3 with the 6-amino group of AppNHp (Figure 4B). While the side chain of the E175 residue in the G-loop of IRAK3 does not have fully defined electron density, chemical bond and Ramachandran restraints of the peptide backbone indicate the most-favorable rotamer of E175 to protrude into the ATP-binding pocket. Interestingly, superposition with AppNHp-bound IRAK4 shows that the modeled E175 side chain is in close proximity with the γ-phosphate of AppNHp (Figure 4B). We predict that the rigidification of the IRAK3 G-loop by the hydrophobic F177 anchor further stabilizes the orientation of E175. Indeed, similar aspartate-substitutions of G-loop residues have been shown to cause negative charge repulsion between the phosphate- and carboxyl-groups and were used to generate ATP-binding-deficient kinase mutants (Zeqiraj et al., 2009a).

In contrast, superposition of IRAK3 with IRAK4 bound to the *pan*-kinase inhibitor staurosporine indicates no steric clashes between staurosporine and IRAK3 (Figure 4C). The distance between L225 and the closest atom of staurosporine is 2.8 Å, while the carboxyl-group of E175 is 6.6 Å away and not in range to interact with any part of the staurosporine molecule. Together, these observations suggest that the ATP-binding pocket of IRAK3 might be unfavorable for binding of ATP but would allow staurosporine binding.

To test this prediction, we used thermal-shift assays with the pseudokinase domains of IRAK2 and IRAK3 following addition of MgCl_2_ (500 μM), ATP (50 μM), MgCl_2_ and ATP (500 μM, 50 μM) or staurosporine (10 μM) (Figures 4D and 4E). In agreement with the structure-guided predictions, MgCl_2_ and ATP did not induce significant thermal shifts in IRAK3, while the addition of staurosporine resulted in a +7.9°C shift of the melting temperature. In comparison, IRAK2 showed robust thermal stabilization with both Mg-ATP and staurosporine. These results are supported by a study that classified 31 different pseudokinase domains on the basis of their nucleotide-binding behavior using thermal-shift assays, which reported a lack of ATP-binding for IRAK3, but robust induction of thermal shifts by the *pan*-kinase inhibitors VI16832 and DAP (Murphy et al., 2014).

Intracellular ATP levels are typically in the mM concentration range (Kennedy et al., 1999). We therefore performed a dose-response analysis, where ATP concentrations were varied from 0 to 1.5 mM, to determine whether IRAK3 would bind ATP at near physiological concentrations. Isothermal analysis of a series of thermal-shift assays with IRAK2 and IRAK3, revealed that IRAK2 binds ATP with a K_d_ of 9.8 ± 3.8 μM at 45°C (Figure 4F), while IRAK3 is a weak ATP-binder with an estimated K_d_ of 196 ± 19 μM at 45°C (Figure 4G). We have also confirmed the weak ATP-binding affinity of IRAK3 using ITC and TNP-ATP saturation binding experiments (data not shown). Collectively, our results therefore suggest that the ATP-binding pocket of IRAK3 may be nucleotide-bound at cellular ATP concentrations.

### IRAK3 Forms an Atypical Head-to-Head Dimer

**Figure 5:**
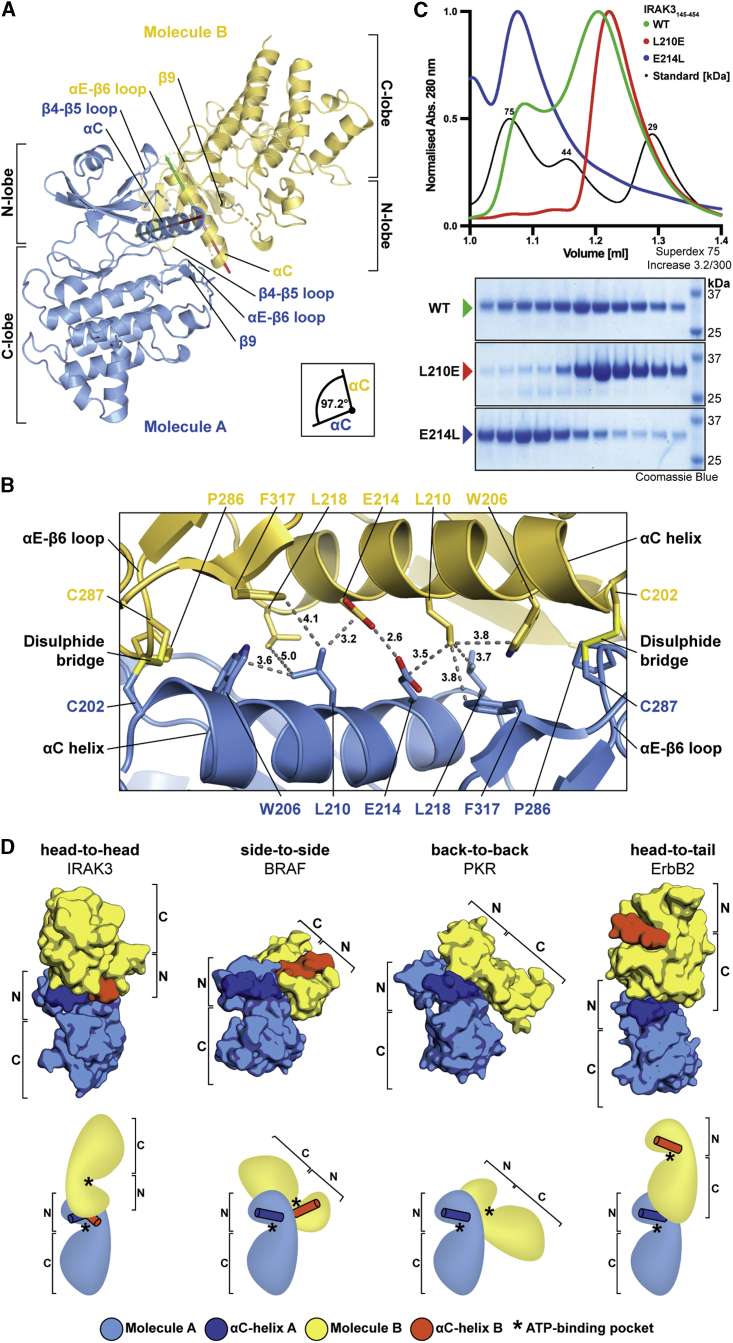
IRAK3 Forms Homodimer in Solution and in Crystal Structure (A) IRAK3 homodimer with monomers in blue and yellow and highlighted regions that contribute to the dimer interface. Arrows indicate orientation of αC-helices from N- to C-terminal residues (red to green). (B) Close-up view of IRAK3 “head-to-head” dimer with interface residues shown as stick models. Dotted gray lines indicate distances with annotation in Å. (C) Analytical gel filtration chromatograms of IRAK3_145-454_ wild-type (WT), L210E, and E214L mutants in green, red and blue, respectively. Molecular mass standards in black with conalbumin (75 kDa), ovalbumin (44 kDa) and carbonic anhydrase (29 kDa). Below, SDS-PAGE analysis of fractions collected from analytical gel filtration runs and molecular mass markers in far-right lane. (D) Smooth surface and schematic representations of kinase dimers of IRAK3, BRAF (PDB 3Q4C; Xie et al., 2009), PKR (PDB 2A19; Dar et al., 2005), and ErbB2 (PDB 3PP0; Aertgeerts et al., 2011). Molecules A in light blue with αC-helices in dark blue, molecules B in yellow with αC-helices in orange. N and C indicate N-lobe and C-lobe, respectively. Asterisks indicate location of ATP-binding pockets. See also Figures S2 and S3.

We analyzed the arrangement of the three IRAK molecules in the ASU (chain IDs: A, B, C), which revealed that molecules A and B form a dimer (A-B) (Figure 5A). Molecule C forms a dimer with a symmetry-related molecule by a 2-fold rotation, C’ (C-C′) (Figure S2A). The dimers are formed via a pseudosymmetric “head-to-head” arrangement with the αC-helices as central elements of the dimer interface. The αC-helices are rotated by 97.2° clockwise against each other and form interactions with the αC-helix, αE-β6-loop, β4-β5-loop and β9-strand of the opposing molecule. Quaternary structure analysis revealed a buried surface area of 955.6 Å^2^ and 1119.3 Å^2^ between the A-B and C-C′ dimers, respectively. The solvation free energy gain Δ^i^G upon formation was calculated in QtPISA as −24.6 kcal/mol for the A-B dimer and as −27.5 kcal/mol for the C-C′ dimer, with Δ^i^G p values of 0.030 and 0.007, respectively. The large negative Δ^i^G values indicate a strong positive protein affinity caused by hydrophobic interactions, while the low p values imply a high specificity of the interfaces and a low likelihood of being an artifact of crystal packing. Interestingly, three dimers (A-B, C-C′, A′-B′) assemble to form a higher-order helical hexamer (B-A-C-C′-A′-B′) in the crystal via C-lobal interfaces with central αG-helices between A-C and C′-A′ molecules (Figure S2).

The head-to-head dimer interface of IRAK3 is made up of a network of hydrophobic interactions with W206, L210, L213, L217, L218, and F219 in the αC-helix of one IRAK3 molecule interacting with Y230 and T232 of the β5-strand, P286 and C287 of the αE-β6-loop, and F317 of the β9-strand of the other IRAK3 molecule in the dimer (Figures 5B and S3A). The interface is flanked by two disulfide bonds formed between C202 at the start of the αC-helices and C287 of the αE-β6-loops. Strikingly, E214 of both αC-helices is located at the center of the interface at a distance of 2.6 Å from each other, which suggests hydrogen-bonding between the carbonyl- and hydroxyl-groups of the opposing glutamate side chains.

Conservation analysis of the αC-helix residues revealed that many of the key residues of the head-to-head interface are highly conserved through vertebrate evolution, including W206, L210, L213, E214, L217, L218, and F219 (Figure S3B). One of the disulphide-bond forming cysteines, C287 in human IRAK3, is highly conserved across vertebrates. In contrast, the position of the other cysteine, C202, is less conserved but prefers cysteine when present (Figure S3B). Intriguingly, most species that lack the cysteine equivalent to C202, for example rodents and bony fish, harbor an additional cysteine predicted to be within or close to the head-to-head interface and might participate in disulphide-bridging. In rodents, an extra cysteine is located N-terminal of the pseudokinase domain, while in bony fish, a cysteine is found at the position of human Y230 in the β5-strand that directly contributes to the head-to-head dimer interface (Figure S3C). In addition, the lack of cysteine residues in equivalent positions in the other human IRAK family members suggests that disulphide-bond formation is specific for IRAK3 and that IRAK3 dimerization could be a redox-regulated event.

To validate the head-to-head dimers observed in the crystal structure, we performed analytical size exclusion chromatography, which revealed that IRAK3 exists in a dynamic equilibrium between monomeric and dimeric states under reducing conditions (Figure S3D). Next, we introduced structure-guided mutations in the IRAK3 homodimer interface to confirm that the in-solution dimer is identical to the head-to-head dimer observed in the crystal structure. We chose the central L210 and E214 residues to generate dimer-disrupting and dimer-stabilizing mutations, respectively. The L210 residue of one IRAK3 molecule forms hydrophobic interactions with the opposing IRAK3 via the γ- and δ-carbons of E214 and the sidechain of L218 (Figure 5B). We predicted that a L210E substitution would break these hydrophobic interactions and in addition introduce a negative charge that would repulse E214 of the opposing molecule, thereby disrupting the IRAK3 dimer. Conversely, we predicted the unusual proximity of the two E214 residues in the dimer interface to be energetically unfavorable due to Coulomb repulsion of the negatively charged glutamate residues. Hence, the introduction of an E214L mutation would remove the repulsive force and strengthen the hydrophobic interaction at the dimer interface, thereby leading to a stabilization of the IRAK3 dimer.

To test these structure-based predictions, the dimerization behavior of wild-type and mutant IRAK3 pseudokinase domains were analyzed by analytical size exclusion chromatography (Figure 5C). The wild-type IRAK3 elutes as two peaks corresponding to the dimeric and monomeric forms, respectively. The dimer-disrupting L210E point-mutant of IRAK3 was observed as a monomer, while the E214L mutant eluted as a dimer. This experiment therefore confirms that the head-to-head dimer, formed between the IRAK3 molecules A-B and C-C′ in the crystal, is identical to the dimer observed in solution. Further, the L210E mutant was identified as a dimer-disrupting mutation and E214L as a dimer-stabilizing point-mutant.

Importantly, the formation of the homodimer of IRAK3 is distinct from the three different modes of dimerization described previously to be involved in the allosteric regulation of kinases through direct stabilization of the alpha-C helix (Figure 5D). The RAF family of kinases dimerize in a “side-to-side” fashion, while the eIF2α-related kinases form “back-to-back” dimers and the EGFR kinases are arranged as “head-to-tail” dimers (Lavoie et al., 2014). In comparison, the pseudokinase RNase L forms “back-to-back” dimers (Han et al., 2014), while the heterodimeric complex of the pseudokinase HER3 with EGFR arranges in a “head-to-tail” fashion (Littlefield et al., 2014). Outside of this classification, pseudokinases have been observed to dimerize through adjacent N- or C-terminal domains, as seen for the pseudokinase SgK223 (Patel et al., 2017), through additional auxiliary proteins, as in the trimeric complex of the pseudokinase STRADα with LKB1 and Mo25 (Zeqiraj et al., 2009b), or through kinase:substrate-like complexes with interactions mainly through the C-lobal subdomains, as reported for the MLKL-RIPK3 and KSR-MEK complexes (Xie et al., 2013; Lavoie et al., 2018).The IRAK3 dimer we identify here uses a previously unidentified interface centered on the αC-helices, which represents a fourth mode of “head-to-head” dimerization.

### Asthma-Associated Mutations Locate to an Evolutionarily Conserved Surface of IRAK3 that Resembles the IRAK4 Homodimer Interface

**Figure 6:**
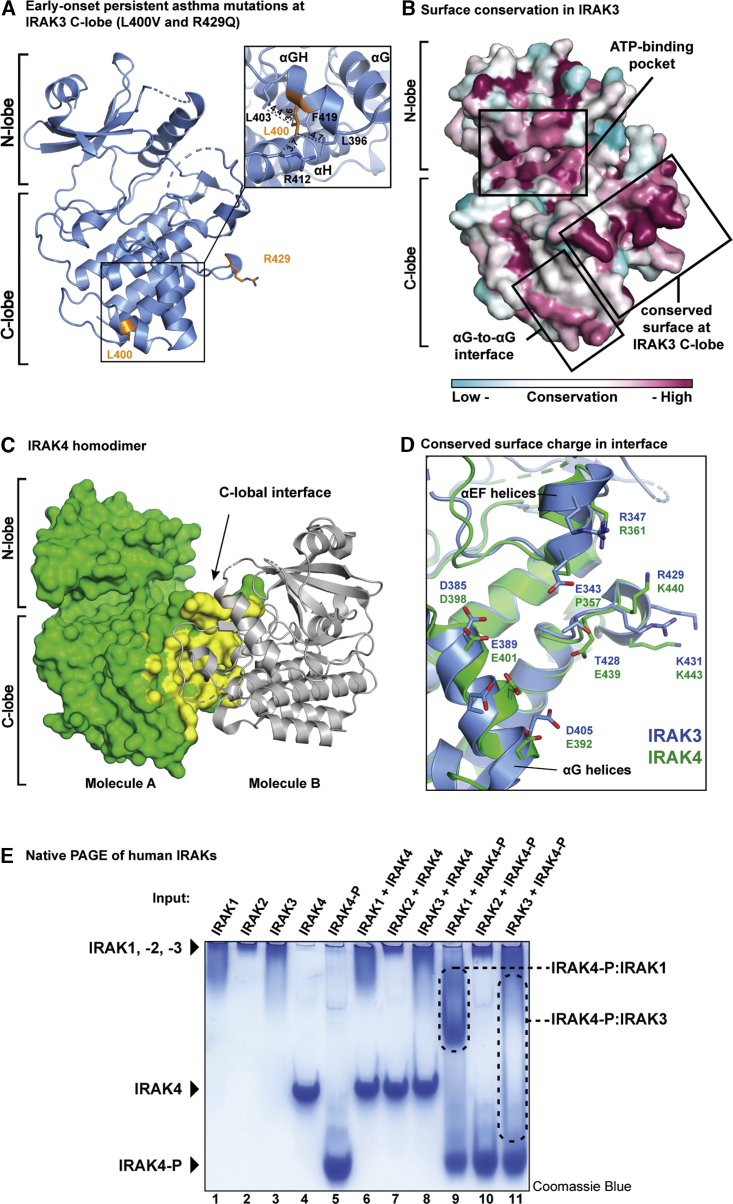
Evolutionarily Conserved Residues and Disease-Associated Mutations of IRAK3 Locate to C-lobe and Superimpose to C-lobal Dimer-Interface of IRAK4 (A) Location of IRAK3 mutations associated to early-onset persistent asthma on pseudokinase domain. (B) IRAK3 surface colored by conservation in vertebrate orthologues from low (green) to high (red). (C) IRAK4 homodimer (PDB 4U97) with molecule A as green surface and oriented as IRAK3 in (B). The second IRAK4 molecule B is shown as gray ribbon. The dimer interface on molecule A is highlighted in yellow. (D) Superposition of IRAK3 (blue) and IRAK4 (green; PDB 4U97) with close-up view of central C-lobal dimer interface residues as stick models. (E) Native PAGE co-migration analysis of the kinase domain of IRAK4 and phosphorylated IRAK4 (IRAK4-P) with kinase and pseudokinase domains of IRAKs-1, -2 and -3. Gel stained with Coomassie Blue. Inputs are indicated above numbered lanes and complexes of IRAK4-P with IRAK1 and IRAK3 are boxed by dotted lines.

Two of the mutations in IRAK3 that are linked to the pathogenesis of early-onset persistent asthma, L400V and R429Q, reside within the pseudokinase domain and both locate to the C-lobe (dbSNP ID: rs146120640 and rs140671957; Balaci et al., 2007). L400 is located at the solvent interface of the αGH-helix and interacts with the neighboring L396, L403, R412 and F419 residues (Figure 6A). The L400V mutation might therefore shift the position of the αGH- and adjacent αG-helices. The R429 residue is solvent exposed and located in the loop connecting the αH- and αI-helices. The R429Q mutation would therefore change the electrostatic surface potential of this region.

To gain insights into these disease-associated mutations, we analyzed the surface conservation of human IRAK3 based on sequence conservation in vertebrate IRAK3 orthologues, which revealed regions of conservation that are not evident from the primary sequence alone. Two conserved surface areas of IRAK3 are the ATP-binding pocket and the head-to-head dimer interface (Figure 6B and S4A). In addition, we identified a third region of high conservation in the C-lobe of IRAK3 between the αEF- and αG-helices (Figure 6B). Strikingly, both residues of the asthma-associated mutations in IRAK3 lie within or are directly adjacent to this conserved surface. We further noticed that the conserved C-lobe surface in IRAK3 closely resembles the C-lobal homodimer interface of unphosphorylated IRAK4 (Figure 6C; PDB 4U97). Structural alignment of IRAK3 and IRAK4 showed a conservation of surface charges of most residues at the center of this C-lobe interface (Figure 6D). Of note, mutation of K440 to E in IRAK4, the residue equivalent to R429 in IRAK3, was shown to disrupt the IRAK4 homodimer (Ferrao et al., 2014).

Based on these observations, we postulated that IRAK3 and IRAK4 may interact via their pseudokinase and kinase domains to form a higher-order IRAK3-IRAK4 complex. To further test this possibility, we performed a native PAGE co-migration analysis with the kinase and pseudokinase domains of IRAK family members (Figure 6E). The kinase and pseudokinase domains of IRAK-1, -2, and -3 have basic theoretical isoelectric points of ∼8 and therefore migrate slowly in a native gel at pH 8.8 (lane 1–3). In contrast, the kinase domain of IRAK4 is more acidic with a theoretical isoelectric point (pI) of ∼5 and migrates more rapidly during native PAGE in its unphosphorylated and phosphorylated (IRAK4-P) forms (lane 4–5).

We did not observe co-migration of unphosphorylated IRAK4 with any of the other IRAK family members (lane 6–8). However, the kinase domains of IRAK1 and IRAK4-P did co-migrate, indicating the formation of a stable complex (lane 9). This result agrees with previous reports by Wang and colleagues who showed co-migration of the kinase domain of IRAK1 with full-length phosphorylated IRAK4 (Wang et al., 2017). Our findings therefore establish that the kinase domains of IRAK1 and phosphorylated IRAK4 are sufficient for this complex to form, which might underlie the proposed allosteric activation of IRAK1 by IRAK4 (Vollmer et al., 2017). In contrast, IRAK2 did not co-migrate with IRAK4-P (lane 10). In the case of IRAK3, elongated bands of IRAK3 and IRAK4-P indicate a weak interaction that causes IRAK3 to be dragged into the native gel, while delaying migration of IRAK4-P (lane 11). This indicates the formation of a transient complex between the pseudokinase domain of IRAK3 and the phosphorylated kinase domain of IRAK4. Full-length IRAK family members are thought to be recruited via their N-terminal DDs into the oligomeric Myddosome complex (Motshwene et al., 2009; Lin et al., 2010), leading to an increased local concentration of the kinase and pseudokinase domains. Within this complex, the transient interaction between IRAK3 and phosphorylated IRAK4, even if of weak affinity, may become functionally relevant.

**Figure 7:**
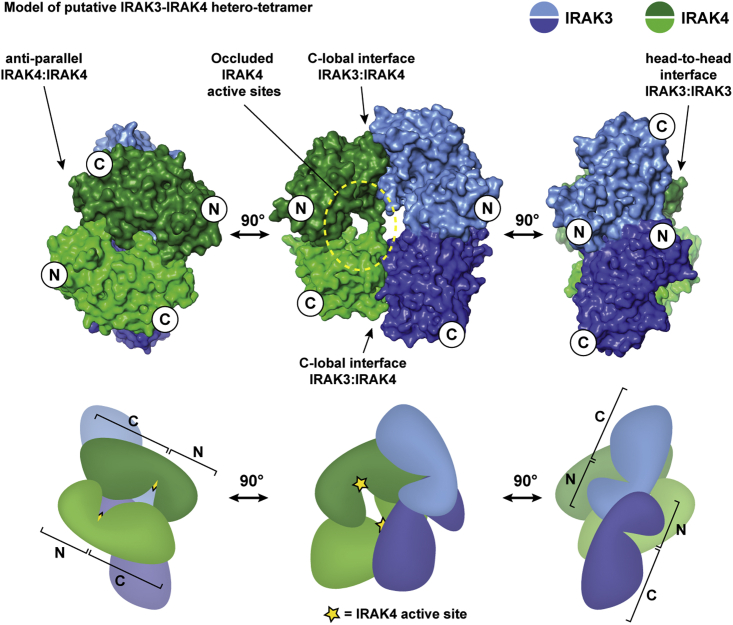
Model of Putative IRAK3-IRAK4 Hetero-Oligomerization Center top: Superposition of IRAK3 dimer and IRAK4 (PDB 4U97) with C-lobal interface between IRAK3 and IRAK4 molecules in blue/purple and green/dark green, respectively. Model rotated by 90° along z axis to the left and right. Arrows and yellow dotted circle indicate interaction interfaces and occluded IRAK4 active sites, respectively. Below, schematic representation of hetero-tetrameric assembly with N- and C-lobes indicated as N and C. Location of IRAK4 active site is marked by a yellow star. See also Figure S4.

Structural superposition of the IRAK3 homodimer with IRAK4 homodimers revealed that the respective head-to-head and C-lobal dimerization interfaces are not overlapping and could therefore co-exist within one complex (Figure 7). This assembly arranges the two kinase domains of IRAK4 in an anti-parallel orientation to each other with compatible electrostatic surface potentials (Figures 7 and S4B). Notably, this arrangement buries the active sites of IRAK4 within the complex and it is tempting to speculate that the pseudokinase domain of IRAK3 might interact with the kinase domain of IRAK4 to inhibit IRAK4 catalytic activity.

Strikingly, the aforementioned helical hexamer of IRAK3 formed in the IRAK3 crystal is also compatible with the C-lobal IRAK4 interface. Superimposition of IRAK4 dimers onto each monomer of the IRAK3 hexamer creates an oligomeric assembly where IRAK4 molecules fill the center of the helical IRAK3 hexamer (Figure S4C). Importantly, the N- and C-termini of the pseudokinase and kinase domains are solvent exposed in both models of tetrameric and oligomeric assemblies and would therefore be able to accommodate the additional N- and C-terminal domains of full-length IRAK3 and IRAK4.

## Discussion

Most canonical protein kinases can switch between an active, closed conformation and an unproductive, open conformation, and employ various regulatory mechanisms that control this transition (Kornev et al., 2006; Endicott et al., 2012). A special type of allosteric regulation of kinase activity is mediated by heterodimerization between kinases and pseudokinases. Like their active kinase counterparts, many pseudokinases have retained their ability to bind ATP and to switch between open and closed conformations (Boudeau et al., 2006; Zeqiraj and van Aalten, 2010; Dar, 2013). For example, ATP-binding to the pseudokinase STRADα assists in stabilization of a pseudoactive, closed conformation, which in turn allows STRADα to bind to and activate the LKB1 kinase (Zeqiraj et al., 2009a). Here, we find that IRAK3 has a weak affinity for ATP, despite having a mostly unobstructed ATP-binding pocket that is accessible for the ATP-competitive inhibitor staurosporine. The only other pseudokinase described to have similar binding behavior is the JH2 pseudokinase domain of TYK2, which has a low affinity for ATP, but is still capable of binding to ATP-competitive inhibitors (Murphy et al., 2014; Min et al., 2015). This characteristic of IRAK3 makes it an attractive candidate to develop potent inhibitors that would compete successfully with the high intracellular ATP levels present *in vivo*. Such inhibitors could either stabilize the closed conformation or destabilize it to affect the downstream signaling events that IRAK3 regulates. A similar approach has been established for small molecules that disrupt the conformation of the pseudokinase ROR1 (Sheetz et al., 2020). Alternatively, inhibitors could be used to develop PROTACs for targeted degradation, as has been demonstrated recently (Degorce et al., 2020).

The IRAK3 structure revealed an evolutionarily conserved cysteine in the R-spine that stabilizes the closed conformation. This suggests that the conformation of IRAK3 might be regulated by a redox-based mechanism, where oxidation or another modification of this cysteine would prevent R-spine assembly, thereby preserving an open conformation. The presence of two disulfide bridges that stabilize the interface of the head-to-head dimer of IRAK3 suggests that IRAK3 might be additionally subject to redox-regulation. Indeed, reactive nitrogen and oxygen species are reported to have dual roles in innate immunity by acting as immunotoxins and modulators of immune signaling (Van Dervort et al., 1994; Jacobs and Ignarro, 2003; Grumbach et al., 2005; Li and Engelhardt, 2006; Wink et al., 2011). Direct regulation of protein kinases by thiol-modifications has been reported for a number of kinases and the high prevalence of cysteine residues in kinase active sites suggests that it is a commonly used to regulate this “cysteinome” subsection of the kinome (Byrne et al., 2017). Redox-mediated inactivation through modifications of cysteines in the activation segment has been reported for the IKK complex (Reynaert et al., 2006) and more recently for the kinase Aurora A (Byrne et al., 2020). Similarly, redox-mediated activation and inactivation of kinases through intermolecular disulphide-bond formation has been shown for PKA (Brennan et al., 2006) and fructosamine-3-kinases (Shrestha et al., 2020), respectively. Our data suggest that IRAK3 may respond to changes in redox levels in two ways: (1) to transition from a closed to an open conformation through modification of the conserved R-spine cysteine, and (2) to stabilize the dimeric state by forming two disulfide bonds across the dimer interface. Further research is needed to validate and evaluate the redox-sensitivity of IRAK3 and its effects on downstream signaling.

The IRAK3 dimer has a head-to-head dimerization interface, distinct from the three modes of kinase dimerization described previously (Lavoie et al., 2014). The unique interdependent packing of the two αC-helices in the IRAK3 dimer stabilizes the closed conformation. Such stabilization has been observed previously for both kinases and pseudokinases. For example, binding of cyclin A to the kinase Cdk2 leads to its activation due to conformational rearrangements in the active site when cyclin A docks on to the αC-helix of Cdk2. In the absence of cyclin A, the αC-helix is displaced, its hydrophobic spine is broken and Cdk2 remains inactive (Jeffrey et al., 1995; Endicott et al., 2012).

Homo- and heterodimerization with other kinases or pseudokinases is a specialized form of kinase regulation, which has been observed in many kinases. It is typically mediated by *trans*-phosphorylation events, where one kinase phosphorylates the activation loop of the other, or via an allosteric mechanism in which an inhibitory element is displaced or the active site remodeled into a catalytically functional state (Endicott et al., 2012; Lavoie et al., 2014). The homo- and heterodimerization of the kinase domains of IRAK1 and IRAK4 has emerged as an important way in which the Myddosome initiates signal transduction (Kuglstatter et al., 2007; Ferrao et al., 2014; Wang et al., 2017). The unphosphorylated kinase domain of IRAK4 forms a dimer that is primed for *trans*-autophosphorylation, an event that is induced by Myddosome assembly in cells. Once IRAK4 is autophosphorylated, the IRAK4 homodimer dissociates (Ferrao et al., 2014; Wang et al., 2019) and indeed the phosphorylated IRAK4 kinase domain was crystallized as a monomer (Kuglstatter et al., 2007). The kinase domain of IRAK1 forms a stable complex with autophosphorylated full-length IRAK4 (Wang et al., 2017) and we showed here that the phosphorylated kinase domain of IRAK4 is sufficient for this interaction.

The model of IRAK3-IRAK4 hetero-oligomerization we propose here reveals surprisingly compatible interfaces between IRAK3 and IRAK4, as well as an anti-parallel arrangement of IRAK4 molecules that occludes the active site. Importantly, such hetero-oligomeric assembly would be functionally relevant as IRAK4 activity would be inhibited by its interaction with IRAK3 dimers. Although this hypothesis is unproven, our findings lay the foundation for a more detailed analysis of the function of IRAK3 in IL-1R/TLR-signaling, which is still unclear. Interestingly, although IRAK3 expression is low in non-immune cells, it is expressed at similar levels to IRAK4 in mouse bone marrow-derived macrophages and at far higher levels than IRAK1 and IRAK2 (JSC Arthur, Personal Communication, 2020; Figure S4D). IRAK3 and IRAK4 are therefore likely to be major components of Myddosomes in these immune cells. Further studies of the interactions between the kinase and pseudokinase domains of IRAKs will be needed to understand how they regulate each other. We believe that the high concentration of IRAKs 3 and 4 in Myddosomes is likely to favor interactions between their kinase and pseudokinase domains, enabling IRAK3 to modulate the eventual signaling outcome.

## STAR★Methods

### Key Resources Table

**Table:** 

REAGENT or RESOURCE	SOURCE	IDENTIFIER
**Bacterial and Virus Strains**		

DH10EMBacY	Geneva Biotech	N/A

**Chemicals, Peptides, and Recombinant Proteins**		

4-(2-aminoethyl) benzene sulphonyl fluoride hydrochloride (AEBSF)	Apollo Scientific	Cat#BIMB2003
Benzamidine	Sigma Aldrich	Cat#B6506
Dithiothreitol	Melford	Cat#D11000
Sf-900 II SFM insect cell medium	Thermo Fisher Scientific	Cat#10902096
Antibiotic / Antimycotic solution (100 x)	Thermo Fisher Scientific	Cat#15240062
GSH 4B sepharose	GE Healthcare	Cat#17-0756-01
Ni-NTA agarose	Qiagen	Cat#30210
Adenosine 5’-triphosphate	Calbiochem	Cat#1191
Staurosporine	Calbiochem	Cat#569397
Glycerol	VWR	Cat#24388.320
SYPRO orange (5000x)	Sigma Aldrich	Cat#S5692
4-(2-Hydroxyethyl) piperazine-1-ethanesulphonic acid (HEPES)	Sigma Aldrich	Cat#H3375
2-Mercaptoethanol	Sigma Aldrich	Cat#M3148
Magnesium chloride	Sigma Aldrich	Cat#M1028
Seed Bead	Hampton Research	Cat#HR2-320
Seeding Tool	Hampton Research	Cat#HR8-133
PreScission Protease (GST tagged)	MRC PPU Reagents	Cat#GNNYH4
IRAK4 1 - 460	MRC PPU Reagents	Cat#AAH13316

**Experimental Models: Organisms/Strains**		

Sf21 insect cells	Invitrogen	Cat#11497013

**Recombinant DNA**		

6His-TEV-GST-3C-AVI-IRAK1 187-527 D358A	MRC PPU Reagents	Cat#DU56583
6His-TEV-GST-3C-AVI-IRAK2 192-507	MRC PPU Reagents	Cat#DU56367
6His-TEV-GST-3C-IRAK3 145-454	MRC PPU Reagents	Cat#DU56013
6His-TEV-GST-3C-IRAK3 145-454 L210E	MRC PPU Reagents	Cat#DU29610
6His-TEV-GST-3C-IRAK3 145-454 E214L	MRC PPU Reagents	Cat#DU65135
6His-TEV-GST-3C-AVI-IRAK4 160-460 D329A	MRC PPU Reagents	Cat#DU56578

**Deposited Data**		

IRAK3	This paper	PDB 6RUU
PKA	((Knighton et al., 1991)	PDB 2CPK
IRAK4 - AppNHp	(Kuglstatter et al., 2007)	PDB 2OID
IRAK4 - Staurosporine	(Kuglstatter et al., 2007)	PDB 2OIC
IRAK4 – asymmetric dimer	(Ferrao et al., 2014)	PDB 4U97
BRAF	(Xie et al., 2009)	PDB 3Q4C
PKR	(Dar et al., 2005)	PDB 2A19
ErbB2	(Aertgeerts et al., 2011)	PDB 3PP0
CDK2 - ANS	(Betzi et al., 2011)	PDB 3PXF
CDK2 - PKTPKKAKKL	(Bao et al., 2011)	PDB 3QHW
IRβ – Mg-ATP	(Wu et al., 2008)	PDB 3BU5
MLKL	(Murphy et al., 2014b)	PDB 4WMI
TRIB1	(Murphy et al., 2015)	PDB 5CEM
VRK3	(Scheeff et al., 2009)	PDB 2JII

**Software and Algorithms**		

XDS	(Kabsch, 1993)	xds.mpimf-heidelberg.mpg.de
POINTLESS	(Evans, 2006)	www.ccp4.ac.uk
AIMLESS	(Evans and Murshudov, 2013)	www.ccp4.ac.uk
autoPROC	(Vonrhein et al., 2011)	www.globalphasing.com
STARANISO server	(Tickle et al., 2016)	www.globalphasing.com
Phenix software suite	(Afonine et al., 2012)	www.phenix-online.org
BALBES	(Long et al., 2008)	www2.mrc-lmb.cam.ac.uk/groups/murshudov/content/balbes/documentation_fs/man_layout.html#install
MoRDa	(Vagin and Lebedev, 2015)	www.ccp4.ac.uk
COOT	(Emsley et al., 2010)	www2.mrc-lmb.cam.ac.uk/personal/pemsley/coot/
PyMOL	Schrödinger, LLC	Pymol.org
QtPISA	(Krissinel, 2015)	www.ccp4.ac.uk
Clustal Omega	(Goujon et al., 2010)	www.ebi.ac.uk/Tools/msa/clustalo/
WebLogo server	(Crooks et al., 2004)	weblogo.berkeley.edu
ConSurf server	(Ashkenazy et al., 2016)	consurf.tau.ac.il

**Other**		

AKTA pure 25	GE Healthcare	Cat#29-0211-97
Superdex Increase 75 3.2/300 GL	GE Healthcare	Cat#29-1487-23
HiLoad® 16/600 Superdex 75 pg	GE Healthcare	Cat#28-9893-33

### Resource Availability

#### Lead Contact

Further information and requests for resources and reagents should be directed to and will be fulfilled by the Lead Contact, Yogesh Kulathu (y.kulathu@dundee.ac.uk).

#### Materials Availability

Generated cDNA clones are listed in the Key Resources Table and are available through MRC PPU Reagents and Services (https://mrcppureagents.dundee.ac.uk/).

#### Data and Code Availability

The accession number for the coordinates and structure factors of the IRAK3 pseudokinase domain reported in this paper is PDB: 6RUU.

### Experimental Model and Subject Details

All IRAK proteins were produced by expression in Sf21 insect cells (Invitrogen, Cat#11497013). Bacmid DNA was generated by transformation of pFastBac vectors into DH10B EMBacY cells and used for baculovirus generation and amplification in Sf21 cells as previously described (Craig and Berger, 2011). Transformed DH10EMBacY (Geneva Biotech) were grown overnight at 37°C on LB agar supplemented with 10 μg/ml gentamicin, 50 μg/ml kanamycin, 10 μg/ml tetracycline, 0.2 mg/ml Xgal and 1 mM IPTG for blue-white selection. Single white colonies were used to inoculate 5 ml LB medium supplemented with 10 μg/ml gentamicin and 50 μg/ml kanamycin and incubated at 37°C and 180 rpm shaking overnight. Sf21 insect cells were cultured in Sf-900™ II serum-free medium (Thermo Fisher Scientific, Cat# 10902096) at 27°C. Sf21 cells were grown as adherent culture for bacmid transfection and initial virus amplification, and as suspension cultures (110 rpm shaking) for further virus amplification and protein expression.

### Method Details

#### Baculovirus Generation and Protein Production

Expression cultures of baculovirus-infected Sf21 were shaken at 120 rpm for 72-96 h at 27°C until the percentage of YFP-expressing cells reached >80%. At this point, cells were harvested by centrifugation at 2000 g and 4°C for 30 min. Cell pellets were resuspended in ice-cold lysis buffer (50 mM HEPES pH 7.5, 500 mM NaCl, 20% glycerol, 1 mM AEBSF, 1 mM benzamidine, 5 mM 2-mercaptoethanol) and lysed by 30 passes in a Dounce homogeniser on ice. Cell debris was removed by centrifugation at 30,000 g and 4°C for 30 min. Ice-cold buffer A (50 mM HEPES pH 7.5, 500 mM NaCl, 20% glycerol, 5 mM 2-mercaptoethanol) was used for resin equilibration and wash steps during subsequent tandem-affinity purifications. IRAK proteins were purified by an initial pass over Ni-NTA resin, to remove endogenous GSH-binding proteins, and subsequent purification on GSH 4B sepharose. The protein was eluted from GSH-resin by cleavage of the 6His-GST-tag with PreScission protease at 4°C for 4-16 h. Finally, proteins were cleaned up in a gel filtration run with a High Load Superdex 75 pg 16/600 column equilibrated with buffer B (20 mM HEPES pH 7.5, 300 mM NaCl, 10% glycerol, 1 mM DTT).

#### Analytical Size Exclusion Chromatography

Analytical gel filtration runs were performed on a Superdex Increase 3.2/300 column (GE Healthcare) equilibrated with 20 mM HEPES pH 7.5, 200 mM NaCl, 10% glycerol, 1 mM DTT. Sample were loaded in 25 μl volumes at 1-5 mg/ml concentrations. Elution fractions were analysed by SDS-PAGE and stained with Coomassie blue.

#### X-ray Crystallography and Structural Analysis

Small crystal clusters of IRAK3 were initially observed in the commercial crystal screen AmSO4 Suite (Qiagen) and manually optimised to obtain single, diffraction-quality crystals. The best crystallisation results were achieved by mixing 3 μL of purified IRAK3_145-454_, concentrated to 7-10 mg/mL, with 1.5 μL of crystallisation cocktail containing 0.1 M citric acid pH 5.0 and 1.62 M ammonium sulfate (2:1 drop ratio) and streak seeding. A crystal seed stock was obtained by mixing five 3 μl drops containing small IRAK3 crystals with 50 μL of crystallisation cocktail, followed by vortexing for 30 s in a ‘Seed Bead’ tube (Hampton Research) to crush the crystals. Crystal seeds were diluted 1:1000 in crystallisation cocktail and introduced using a ‘Seeding tool’ (Hampton Research) in a smooth motion. After seeding, the drops were incubated at 20°C in hanging drop plates with 1.0 ml reservoir volume and crystals typically appeared within two days and reached maximum size in 5-7 days. This procedure yielded single rod-shaped crystals with dimensions of up to ∼50x50x200 μm alongside multi-crystal clusters.

The IRAK3 crystals displayed relatively weak and anisotropic diffraction properties and the majority of the tested crystals diffracted in the range of 5-8 Å. As estimated by the Matthews coefficient, the IRAK3-PK crystal had a relatively high solvent content of ∼70%. IRAK3 crystals that diffracted to 2.9 Å were soaked for 16 h in crystallisation cocktail containing 5 mM ethyl mercury phosphate (EMP) and 30% glycerol before mounting on a nylon-loop and vitrification in liquid nitrogen. Diffraction data was collected at the i24 beamline at the Diamond Light Source, UK. All tested IRAK3 crystals consistently displayed anisotropic diffraction properties. We therefore processed the diffraction data with XDS (Kabsch, 1993), AIMLESS (Evans and Murshudov, 2013) and POINTLESS (Evans, 2006) followed by anisotropic processing with the Staraniso server (staraniso.globalphasing.org; Tickle et al., 2016) with directional resolution cut-offs determined by local mean I/σ(I) ≥ 1.2 as 2.9 Å, 3.2 Å and 4.1 Å along the reciprocal c-, a-, and b-axes, respectively.

The IRAK3 structure was determined by molecular replacement in BALBES (Long et al., 2008) using a search model generated in MoRDa (CCP4 online) (Vagin and Lebedev, 2015) from the protein kinase Pto from *Solanum pimpinellifolium*, the currant tomato (PDB ID: 2QKW) (Xing et al., 2007). The model was manually build and refined using COOT (Emsley et al., 2010) and phenix.refine (Afonine et al., 2012). Quaternary structure analysis of the IRAK3 interfaces was performed with QtPisa (Krissinel, 2015). Structural superposition and visualisations were created in PyMOL (Schrödinger, LLC) and electrostatic surface potential were generated with the APBS tool 2.1 plugin (Baker et al., 2001).

#### Thermal Shift Assays and Isothermal Analysis of Thermal Shift Data

The target proteins were prepared in 10 μl volumes at 2.5 μM in 20 mM HEPES pH 7.5, 200 mM NaCl, 10% glycerol, 1 mM DTT, 3x SYPRO orange dye and additives on ice subsequently heated in a temperature ramp of 1°C/min from 5°C to 75°C while recording the FRET channel using a CFX384 real-time PCR detection system (BioRad). Thermal shift data was analysed using the ‘DSF analysis v3.0’ Excel script as published by the Structural Genomics Consortium, Oxford (Niesen et al., 2007) and the Boltzmann sigmoidal fitting function in GraphPad Prism v8.0. Isothermal analysis was performed with the ‘DSF fitting’ python program (Bai et al., 2019). The change in heat capacity upon unfolding ΔCp was estimated as 4.03 and 4.27 kcal/K^∗^mol for the pseudokinase domains of IRAK2 and IRAK3, respectively. ΔCp estimation was based on the change in the solvent accessible surface area of nonpolar residues between the folded and unfolded states ΔASA (Figure 3 in Novokhatny and Ingham, 1997). The ASA of folded states of IRAK2 and IRAK3 were calculated in PyMOL using a computational model of IRAK2 generated by one-to-one threading with IRAK4 (PDB 4U97) in Phyre2 (Kelley and Sternberg, 2009) or chain A of the IRAK3 structure, respectively. The ASA of unfolded states were estimated from the primary amino acid sequence with theoretical ASA of individual amino acids (Table 1 in Tien et al., 2013).

#### Multiple Sequence Alignment and Conservation Analysis

Sequences of IRAK3 vertebrate orthologues were retrieved from OrthoDB (Kriventseva et al., 2015) and manually purged for duplicates within species and sequences lacking the pseudokinase domain in JalView (Waterhouse et al., 2009). Sequences of all human kinase domains were retrieved from KinBase (Manning et al., 2002). Sequences were aligned by multiple sequence alignment using Clustal Omega (Goujon et al., 2010) and analysed for spatial conservation by the ConSurf server (Ashkenazy et al., 2016). Sequence conservations were visualised using the WebLogo server (Crooks et al., 2004).

#### Native PAGE Co-migration Assay

Continuous native PAGE 10% acrylamide gels were prepared by polymerisation of 375 mM Tris/HCl pH 8.8, 10% acrylamide (w/v), 0.01% (w/v) ammonium persulfate (APS) by the addition of 0.01% (v/v) TEMED. IRAK4 samples were phosphorylated *in vitro* by incubation of IRAK4_160-460_^D329A^ with 1.5 U active full-length IRAK4 per mg of substrate in the presence of 2 mM ATP, 5 mM MgCl_2_ and 2.5 mM DTT for 2.5 h at 30°C. All native PAGE samples were prepared at a concentration of 1 mg/ml per protein and pre-incubated on ice for 1 h prior to loading. The gels were mounted in a gel running tank with SDS-free native PAGE running buffer (25 mM Tris, 192 mM glycine, pH 8.3). 10 μl sample volumes were loaded and separated by application of a constant electric current of 10 mA for 2 h on ice and stained with Coomassie blue.

### Quantification and Statistical Analysis

Thermal shift assays were conducted in triplicate and isothermal analysis was performed with the ‘DSF fitting’ python program (Bai et al., 2019). The binding constants and standard errors were determined by fitting the data using GraphPad Prism (v8.4.2) as described in Bai et al., 2019 to a nonlinear regression model (Y = 1 / (1 + (1 / Ku) ^∗^ (1 + (((X - P - Kd ^∗^(1 + Ku) + (((P – X + Kd ^∗^ (1 + Ku))ˆ2 + 4 ^∗^ (X ^∗^ Kd + X ^∗^ Kd ^∗^ Ku))ˆ0.5)) / 2) / Kd))), where Y is the fraction of unfolded protein, X is the ligand concentration, Kd is the equilibrium dissociation constant, Ku is the equilibrium constant of the protein unfolding reaction and P the protein concentration in μM).

## Supplemental Information

Document S1. Figures S1–S4 and Table S1

Document S2. Article plus Supplemental Information
