# Supplementary material for: Dimeric Structure of the Pseudokinase IRAK3 Suggests an Allosteric Mechanism for Negative Regulation
Source: Structure. 2021 Mar 4;29(3):238–251.e4. doi: 10.1016/j.str.2020.11.004 (PMC7955167; doi:10.1016/j.str.2020.11.004)
Supplement: Document S1. Figures S1–S4 and Table S1 [file mmc1.pdf]

Structure, Volume *29*

Supplemental Information

Dimeric Structure of the Pseudokinase IRAK3 Suggests an Allosteric Mechanism for Negative Regulation

Sven M. Lange, Marina I. Nelen, Philip Cohen, and Yogesh Kulathu

Supplementary Material

Supplementary Table 1. Data collection and refinement statistics, Related to Figure 2.

Statistics for the highest resolution shell are shown in parentheses.

	**Pseudokinase domain of human IRAK3$_{145-454}$**
**Wavelength**	**1.007**
**Resolution range**	**49.0 - 2.9 (3.3 - 2.9)**
**Space group**	**P 21 21 2**
**Unit cell**	**53.55 167.16 179.51 90 90 90**
**Total reflections**	**631331 (33096)**
**Unique reflections**	**21506 (1072)**
**Multiplicity**	**29.4 (30.9)**
**Completeness (spherical)**	**61.1 (11.2)**
**Completeness (ellipsoidal)**	**92.2 (59.6)**
**Mean I/sigma(I)**	**12.1 (1.8)**
**Wilson B-factor**	**77.91**
**R-merge**	**0.280 (2.410)**
**R-meas**	**0.285 (2.450)**
**R-pim**	**0.05 (0.438)**
**CC1/2**	**0.998 (0.702)**
**CC***	**0.999 (0.908)**
**R-work**	**0.2296 (0.4521)**
**R-free**	**0.2594 (0.3049)**
**CC(work)**	**0.914 (0.386)**
**CC(free)**	**0.850 (0.884)**
**Ramachandran outliers**	**0**
**Molprobity clash score**	**10**

Supplementary Figures.

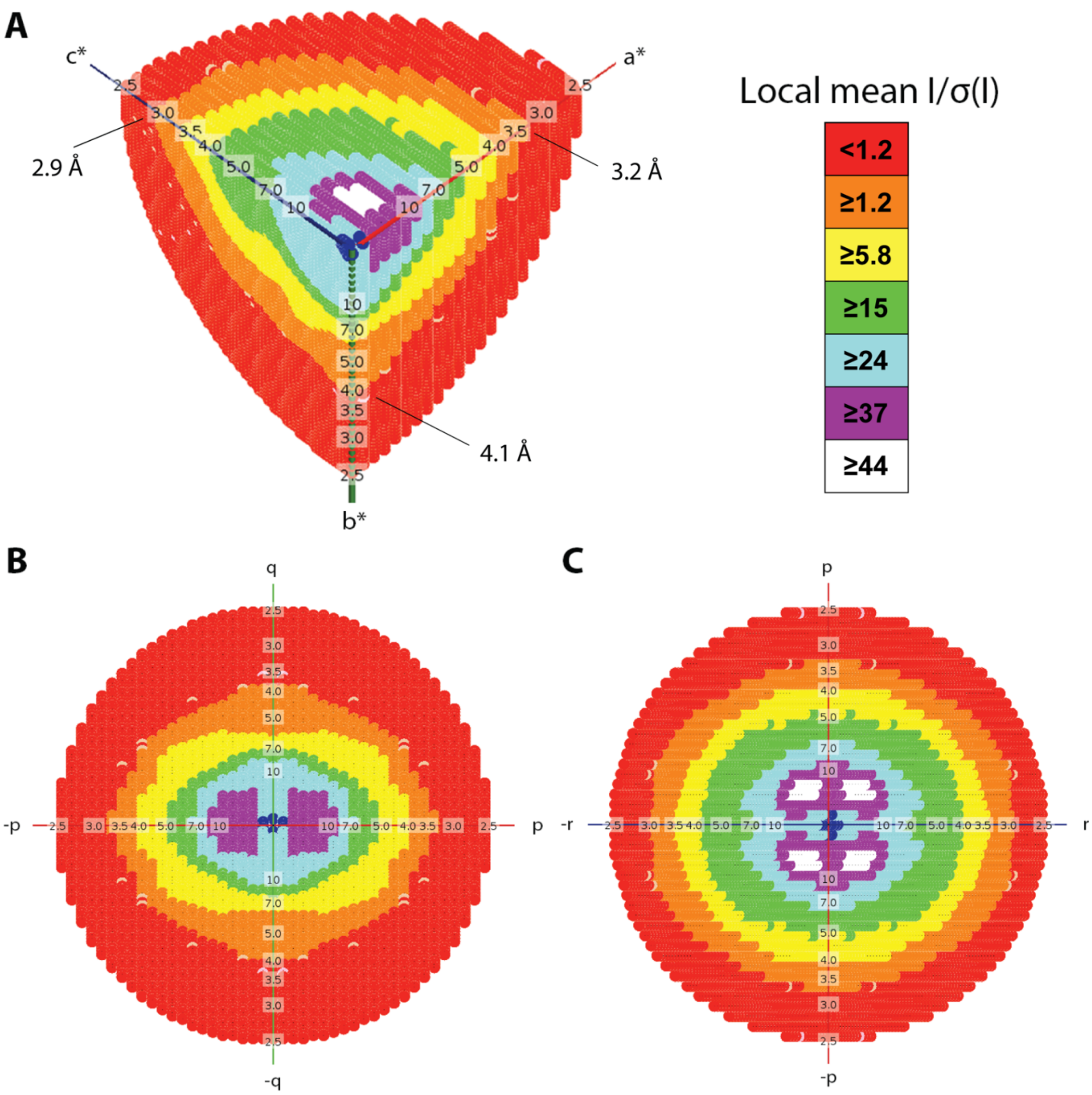

Figure S1. Anisotropic diffraction of IRAK3 crystals, Related to Figure 2.

Visualisation of reciprocal space diffraction data coloured by local I/σ(I) thresholds. (a) Three-dimensional wedge along a*, b* and c* axes. (b, c) Two dimensional views of p0r and pq0 planes of the ellipsoid determined by anisotropic analysis with Staraniso. All axes labels in Å.

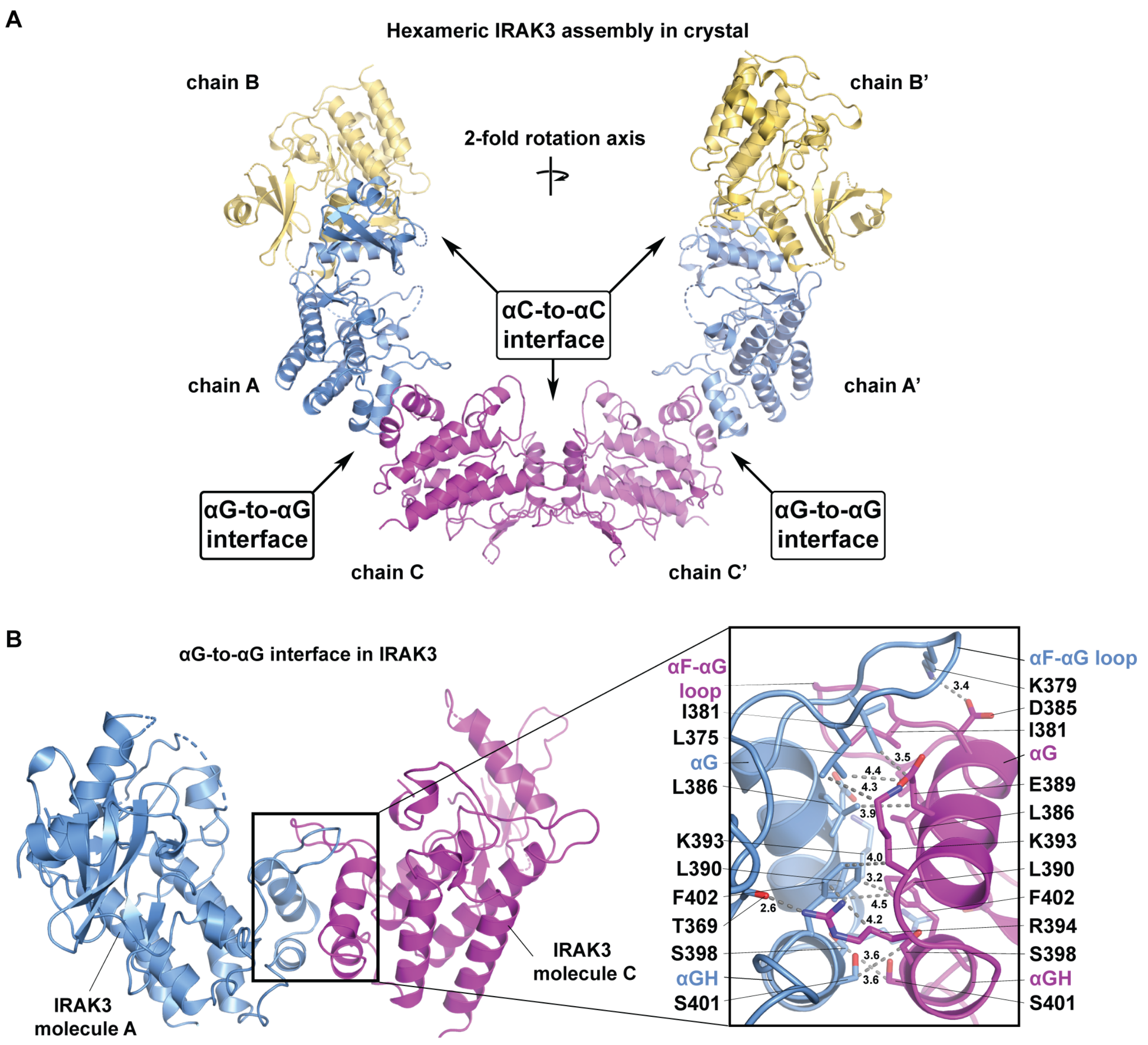

Figure S2. Hexameric assembly of IRAK3 in crystal, Related to Figure 5.

(a) Cartoon representation of the three IRAK3 molecules in the asymmetric unit that form a hexamer with three symmetry-related molecules. Molecules of chain A in blue, chain B in yellow, chain C in purple. Labels indicate αC-to-αC (‘head-to-head’) and αG-to-αG interfaces. (b) Close-up view of αG-to-αG interface with interface residues as stick representation. Grey dashed lines indicate distance measurements between interface residues with labels in Å.

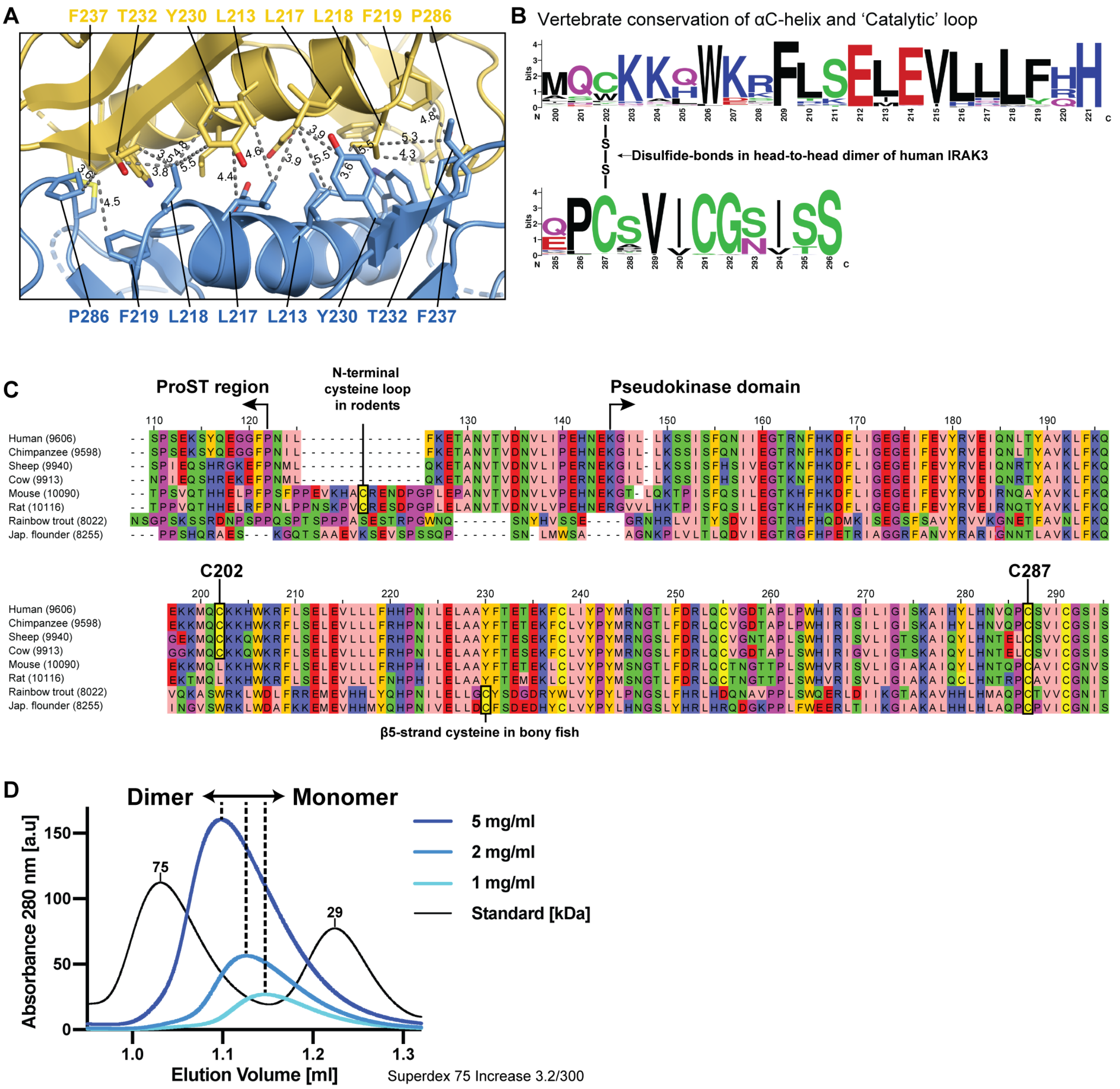

Figure S3. IRAK3 head-to-head dimer and disulphide-bridge conservation, Related to Figure 5.

(a) Back view of head-to-head dimer interface with interface residues as stick models. Grey dotted lines indicate distance measurements with labels in Å. (b) Sequence logos of αC-helix and 'catalytic' loop residues of vertebrate IRAK3 orthologues. Residue numbers of human IRAK3, polar residues in green, non-polar residues in black, basic residues in blue and acidic residues in red. (c) Sequence alignment of selected vertebrates IRAK3 orthologues indicated with unique taxonomy identifiers. Highlighted positions of disulphide-bridge-forming cysteines C202 and C287 in human IRAK3 and

predicted alternative C202 positions at the N-terminal cysteine-loop in rodents and in the β5-strand in bony fish. Residue numbers of human IRAK3 above alignment. Sequence highlighted based on amino acid properties: S/T/N/Q in green, A/V/I/L/M in pink, K/R in blue, D/E in red, G/P in purple, F/Y/W in orange, C in yellow. (d) Analytical gel filtration chromatograms of $IRAK3_{145\text{-}454}$ injected at 5 mg/ml (dark blue), 2 mg/ml (light blue) and 1 mg/ml (cyan). Molecular weight standard in black with conalbumin (75 kDa) and carbonic anhydrase (29 kDa).

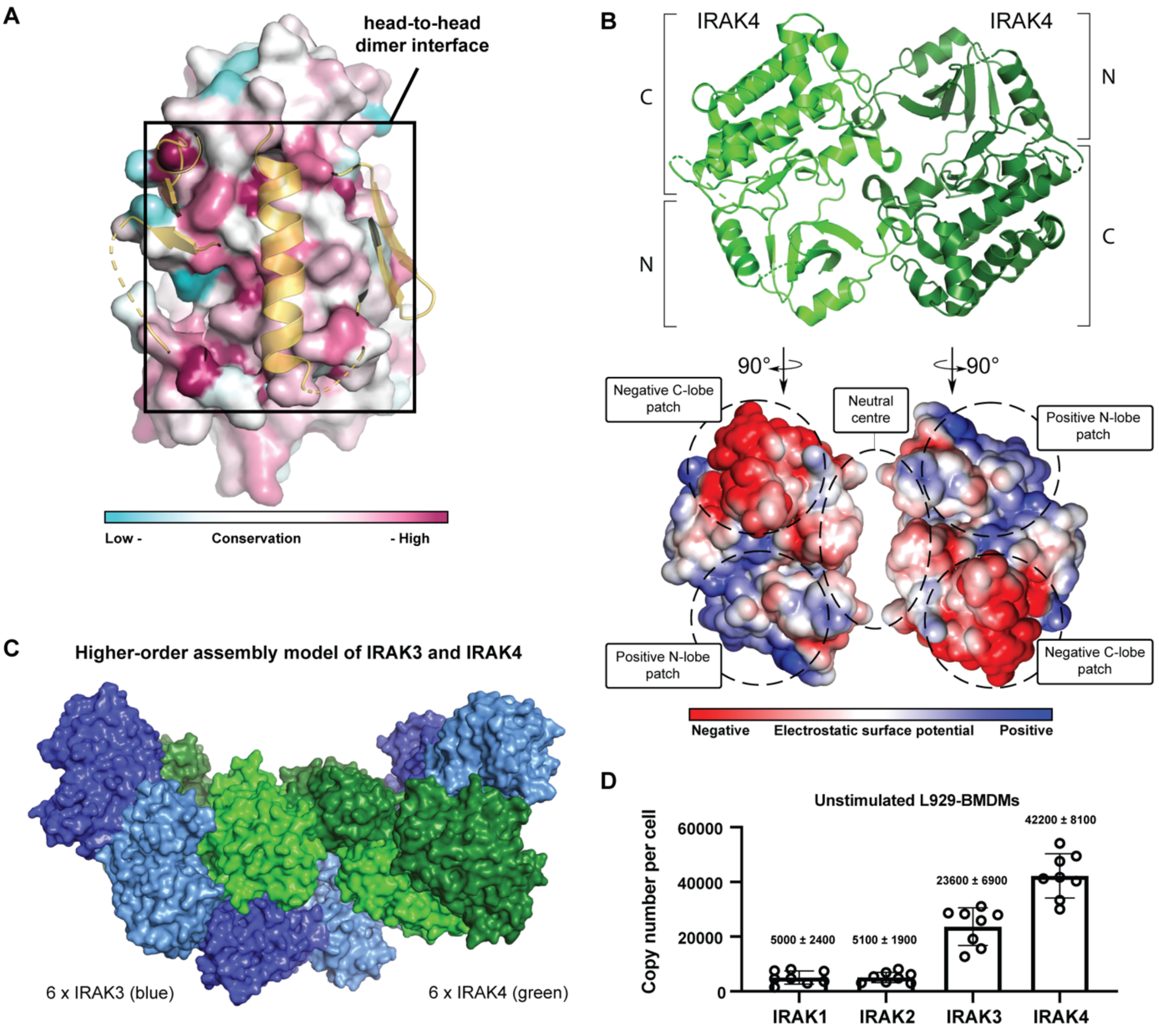

Figure S4. Surface conservation of αC-helix and putative IRAK3-IRAK4 oligomer, Related to Figure 7.

(a) Surface conservation of head-to-head dimer interface with view on surface of molecule A from within molecule B. Surface of molecule A is coloured by conservation in vertebrate orthologues from low (green) to high (red). Molecule B represented as orange cartoon with central αC-helix with view clipped for visibility. (b) Cartoon representation of anti-parallel IRAK4 molecules of hetero-oligomeric assembly with IRAK3. Below, IRAK4 molecules rotated by 90° clockwise and anti-clockwise along z-axis, respectively, and displayed as surface representation coloured by electrostatic surface potential. (c) Model of putative hetero-oligomeric assembly of IRAK3 dimers

(monomers in blue and light blue) with IRAK4 molecules (in green and light green) generated by structural superposition of IRAK3 monomers with IRAK4 homodimers (PDB 4U97). (d) Copy numbers of IRAK molecules per cell in unstimulated L929-mouse bone marrow-derived macrophages obtained by label-free quantitative proteomics. Data processed in MaxQuant and copy number determined by histone ruler method in Perseus. Data represents 4 biological replicates with two technical replicates each. Mean values with SD indicated, error bars represent SD.
